# Pharmacotherapy for Alcohol Use Disorders: Physicians’ Perceptions and Practices

**DOI:** 10.3389/fpsyt.2016.00182

**Published:** 2016-11-14

**Authors:** Caridad Ponce Martinez, Priyanka Vakkalanka, Nassima Ait-Daoud

**Affiliations:** ^1^Department of Psychiatry, Yale School of Medicine, New Haven, CT, USA; ^2^Department of Emergency Medicine, University of Virginia, Charlottesville, VA, USA; ^3^Department of Psychiatry and Neurobehavioral Sciences, University of Virginia, Charlottesville, VA, USA

**Keywords:** alcohol use disorder, pharmacotherapy, family physicians, psychiatrists, survey

## Abstract

**Background and objectives:**

Alcohol use disorders (AUDs) are an important cause of morbidity and mortality. Despite the National Institute on Alcohol Abuse and Alcoholism (NIAAA) recommendations that medications be considered for patients with alcohol dependence, the mainstay of treatment has been counseling. We designed a survey to assess the treatment practices of psychiatrists and family medicine (FM) physicians in an effort to identify barriers to the use of pharmacotherapy and develop strategies to increase physician knowledge and utilization of these medications.

**Methods:**

An anonymous online survey was sent to FM physicians and psychiatrists nationwide. The survey collected demographic information and assessed prescription of medications in treating AUDs, including FDA-approved medications and other medications used off-label for this purpose. We also examined factors that would lead to an increase in AUDs pharmacotherapy.

**Results:**

A total of 491 surveys were completed, with 475 responses included in the final analyses. 45.5% of participants were psychiatrists vs. 54.5% FM physicians. The 74.7% respondents had used medications to treat AUDs, with psychiatrists more likely to have prescribed acamprosate, naltrexone, and several off-label medications. FM physicians were more likely to report efficacy concerns. A majority of all physicians sampled would increase pharmacotherapy of AUDs with increased training.

**Discussion:**

In our sample, most physicians have used medications to treat AUDs. There were concerns about efficacy with all non-FDA-approved medications, but limited treatment success even with FDA-approved medications. Greater education about pharmacotherapy, including predictors for treatment response amongst patients, should help alleviate some of the uncertainties reported with medications’ efficacy and lead to a more individualized treatment approach.

## Introduction

Alcohol-related problems are a vital cause of morbidity and mortality across the world. According to the World Health Organization (WHO), in 2012, about 3.3 million deaths, or 5.9% of all global deaths, were attributable to alcohol use (1). The 2014 National Survey on Drug Use and Health (NSDUH) revealed 23% of people ≥12 years old in the United States (60.9 million) and 6.2% of the population in this age range (16.3 million) reported binge drinking and heavy drinking in the previous 30 days, respectively (2).

Despite the high cost and prevalence of substance use disorders (SUDs), they have historically been undertreated. In fact, according to the 2014 NSDUH, among the 22.5 million individuals older than 12 years who needed treatment for SUDs, only 2.6 million received treatment at a specialty facility (2). Using data from the National Epidemiologic Survey on Alcohol and Related Conditions (NESARC), sponsored by the National Institute on Alcohol Abuse and Alcoholism (NIAAA) in the early 2000s, Hasin and colleagues found that only 24.1% of those with alcohol dependence were ever treated. When compared to treatment rates from data available 10 years earlier, this was actually a decline in treatment rate (3).

The mainstay of treatment for alcohol use disorders (AUDs) in the U.S. has generally been counseling or 12-step programs. Ducharme et al. examined adoption of pharmacotherapy for alcohol dependence in specialty addiction treatment facilities during 1995–2004, finding that this practice was relatively uncommon (4). Federal data from substance abuse treatment facilities across the U.S. from 2013 reveal that only about 17–19% prescribed FDA-approved medications for AUDs treatment (5). Review of prescription practices among substance abuse specialists (6), substance abuse treatment facilities (4), Veterans Health Administration facilities (7, 8), and U.S. sales volume of prescriptions (9) has corroborated these findings.

Since 2005, NIAAA has recommended that medications should be considered for every patient with alcohol dependence (10). There are currently four FDA-approved medications for the treatment of AUDs: disulfiram, naltrexone (oral and extended-release injectable formulations), and acamprosate. Efficacy studies have supported the use of these medications in the treatment of AUDs, with results demonstrating mainly modest effects (11, 12). In a large utilization and cost study of alcohol pharmacotherapy, Baser et al. found that patients who received medication had lower health-care utilization and total costs than patients who did not, despite the costs of these medications (13).

Primary care physicians and psychiatrists have potentially a greater vantage point for detection and perhaps intervention of AUDs. Primary care physicians often identify AUDs when evaluating patients for other medical reasons, while psychiatrists tend to have greater training in addictions and often deal with comorbid psychiatric and SUDs (14). Because prior studies have shown that use of medications for treatment of AUDs varies by medical specialty (6, 9, 14, 15), we designed a survey to assess the practices of the two specialties most likely to detect patients with AUDs, psychiatrists, and family medicine (FM) physicians. This was done in an effort to identify barriers to the use of pharmacotherapy and develop strategies that would lead to greater physician knowledge and ultimately greater use of medications in the treatment of patients with these disorders.

## Materials and Methods

### Participants

Beginning in April 2012, we emailed an online survey designed using SurveyMonkey (https://www.surveymonkey.com), to FM physicians and Psychiatrists at all levels of training nationwide. Participants were recruited by contacting 52 chapters of the American Academy of Family Physicians, 70 chapters of the American Psychiatric Association, and ACGME-accredited psychiatry (183) and FM (455) residency programs in the United States. Invitations to participate were initially sent to the leaders of the specialty chapters, as well as program directors and/or coordinators of the residency programs. They in turn distributed the survey invitation to their professional listservs and their faculty/trainees, respectively. A second email was sent out to all initial contacts, except those who had declined participation, 7–10 days later, as a reminder to complete the survey. Prospective participants were informed that the study’s objective was to assess whether physicians have used medications in the treatment of AUDs and what their experiences have been with these medications. Participants were not compensated for completion of the survey, but were offered the option of entering their email addresses for a raffle of two prizes (an iPad or a 1-year paid membership to the American Society of Addiction Medicine). Responses were collected until mid-June 2012. The University of Virginia’s Institutional Review Board approved the research design.

### Demographic Data

The survey asked physicians’ primary specialty, if they were specialized in addictions, their level of training, or number of years since completion of residency (current resident/fellow, completed residency within last 10 years, completed residency over 10 years ago), their state of practice, and their primary medical practice setting. Participants specializing in both FM and psychiatry as well as those identifying another primary specialization were excluded from this study.

### Survey Tool

The online survey consisted of a total of 24 questions, including demographic questions. Because “skip logic” was employed in some key questions, no participant completed all questions, based on their responses. The questions pertained to participants’ use of pharmacotherapy in the treatment of AUDs, their experiences with several medications, their level of training in pharmacotherapy for AUDs, and factors that would lead to increased prescription of medications in the treatment of AUDs. A definition of AUDs, per DSM-5, was provided. Participants were asked to exclude treatment for alcohol withdrawal in their responses. Survey completion time was about 5 min. All individual responses were kept anonymous.

#### Pharmacotherapy in AUDs

The survey assessed whether participants had prescribed medications in the treatment of AUDs with a yes/no question, which was necessary for further completion of the survey. Because of “skip logic,” participants who selected “no” in this question did not complete the portion of the survey dedicated to evaluation of specific medications. For those who selected “yes,” the medications prescribed were assessed *via* a multiple-choice question that included FDA-approved medications (disulfiram, naltrexone, and acamprosate) as well as several other “off-label” medications obtained from literature reviews – antidepressants, carbamazepine, gabapentin, ondansetron, topiramate, and varenicline (6, 11, 12, 16, 17). A follow-up question asked which of the medications that had been prescribed produced favorable results.

To evaluate participants’ concerns with the prescription of specific medications, we created individual questions regarding naltrexone, disulfiram, acamprosate, topiramate, gabapentin, and varenicline. These included commonly cited reasons for not prescribing these medications, including cost, efficacy, tolerability, and lack of experience with the medication, and poor patient compliance (6, 15).

Participants who indicated they do not prescribe medications in the treatment of AUDs were asked for their reasons in a separate multiple-choice question (i.e., “I do not have patients with alcohol use disorders,” “I prefer to only refer patients to Alcoholics Anonymous or other 12-step programs”).

### Statistical Analysis

Chi-square analyses were used to identify differences by specialization and training level for ever use of medications, medications having the best results, and reasons for not prescribing medications. Univariate analyses of prevalence ratio (PR) estimates of the ever use of these medications were evaluated by specialization and training level, followed by multivariate analyses adjusting for these two variables. Statistical analyses were performed with SAS™ (Version 9.4, SAS Institute, Inc., Cary, NC, USA).

## Results

A total of 491 surveys were completed between April and June 2012.

### Demographics

**Figure 1:**
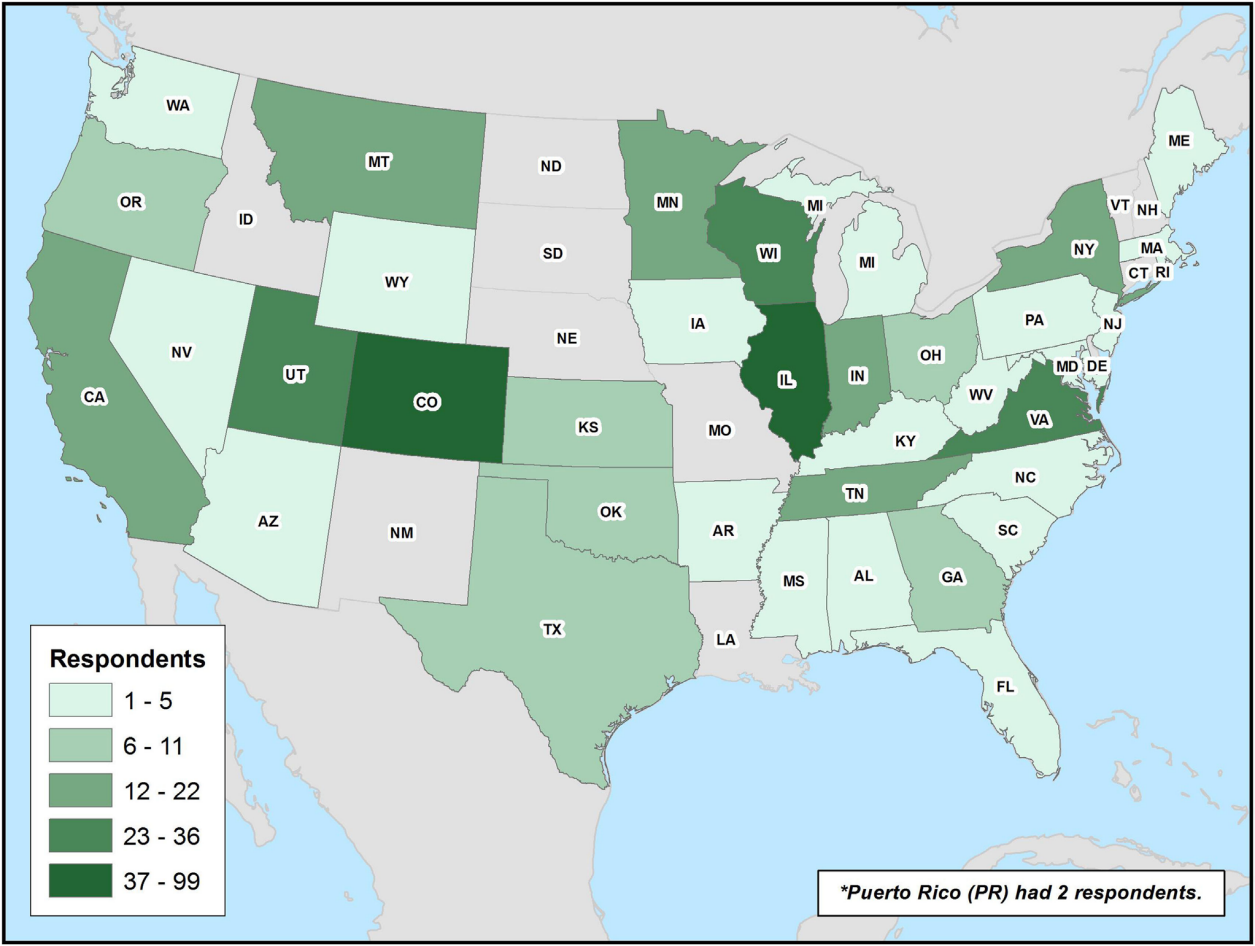
**Geographical distribution of participants**. Survey participants’ geographical distribution by state is provided.

Also, 45.5% of survey participants were identified as psychiatrists vs. 54.5% as FM. A minority of respondents had a subspecialty in addictions (9.9%). The level of training of participants was widely distributed, with 32.2% of participants currently in residency or fellowship training, 20% having completed residency within the previous 10 years, and 47.8% having completed residency >10 years prior. Geographical distribution of respondents is available in Figure 1.

### Pharmacotherapy

While nearly 75% of all participants have ever used medications in the treatment of AUDs, FM participants were less likely to have used pharmacotherapy (*p* < 0.001). After adjusting for level of training, psychiatrists were more likely to have ever prescribed the FDA-approved medications acamprosate (PR: 1.46, 95% CI: 1.21–1.76, *p* < 0.001) and naltrexone (PR: 2.59, 95% CI: 2.07–3.23, *p* < 0.001), respectively (Table 1). Among medications without current FDA approval for treatment of alcohol dependence, psychiatrists were more likely to have ever prescribed carbamazepine (PR: 2.25, 95% CI: 1.32–3.85, *p* = 0.003), gabapentin (PR: 2.84, 95% CI: 1.86–4.34, *p* < 0.001), and topiramate (PR: 4.68, 95% CI: 2.73–8.01, *p* < 0.001). There was no statistical significance between psychiatry and FM in the prescription of antidepressants, ondansetron, and varenicline.

**Table 1:** **Adjusted prevalence ratio of ever use of medications**.

Medication				Training level					
	Psychiatry vs. family medicine^a^			Experience level 2 vs. 1^b^			Experience level 3 vs. 1^c^		
	Adj prev ratio	95% CI	*p* Value	Adj prev ratio	95% CI	*p* Value	Adj prev ratio	95% CI	*p* Value
Acamprosate	1.46	1.21–1.76	<0.001	1.93	1.46–2.53	<0.001	1.46	1.10–1.93	0.009
Carbamazepine	2.25	1.32–3.85	0.003	1.80	0.92–3.55	0.088	1.31	0.70–2.47	0.397
Disulfiram	0.95	0.84–1.07	0.415	1.32	1.04–1.69	0.023	1.51	1.23–1.86	<0.001
Gabapentin	2.84	1.86–4.34	<0.001	1.22	0.81–1.83	0.336	0.77	0.52–1.15	0.202
Naltrexone	2.59	2.07–3.23	<0.001	1.80	1.40–2.31	<0.001	1.51	1.19–1.93	<0.001
Antidepressants (SSRIs/SNRIs)	1.13	0.92–1.39	0.255	1.06	0.81–1.38	0.678	0.86	0.67–1.09	0.215
Ondansetron	0.49	0.26–0.95	0.036	0.35	0.15–0.84	0.019	0.19	0.08–0.41	<0.001
Topiramate	4.68	2.73–8.01	<0.001	1.30	0.83–2.03	0.244	0.94	0.61–1.44	0.766
Varenicline	1.18	0.54–2.56	0.674	1.40	0.52–3.71	0.505	0.72	0.28–1.87	0.505

*^a^Reference group is family medicine*.

*^b^Reference group is current residents/fellows when compared to those completing residency within 10 years*.

*^c^Reference group is current residents/fellows when compared to those completing residency more than 10 years ago*.

*Level 1 = current resident/fellow*.

*Level 2 = completed residency ≤10 years ago*.

*Level 3 = completed residency >10 years ago*.

*Prevalence ratios are adjusted for specialization and level of training*.

*Red font highlights significant p Value*.

There were differences in the ever use of medications to treat AUDs according to level of training. The differences in prescription of acamprosate, disulfiram, naltrexone, and ondansetron were statistically significant across these groups (Table 1). Disulfiram, the first FDA-approved medication for treatment of alcohol dependence, was most frequently prescribed by physicians who completed training >10 years ago (*n* = 150, 80.2% of physicians in that level of training). When compared with current residents/fellows, senior physicians were more likely to have ever prescribed acamprosate, disulfiram, and naltrexone (Table 1).

More than one-third of physicians who had prescribed one of the FDA-approved medications for treatment of AUDs had prescribed at least one of the other FDA-approved medications. For example, 41.7% of physicians had prescribed both disulfiram and acamprosate, 40.0% had prescribed acamprosate and naltrexone, and 42.3% had prescribed disulfiram and naltrexone. Compared to FM, psychiatrists had the best results with naltrexone (47.5 vs. 13.8%) and disulfiram (27.1 vs. 24.7%). FM physicians were more likely to select “none” as their medication of choice (33.9 vs. 20.4%) and acamprosate (20.1 vs. 13.3%). Concerns regarding efficacy also appeared when analyzing the groups according to level of training; 31.6% of physicians who completed training >10 years ago had the best results with disulfiram, vs. 22.1% of physicians who completed training within 10 years and 17.6% of current residents or fellows (*p* = 0.031). Physicians who completed residency within 10 years had the best results with acamprosate (28.6%), vs. 16.0% of more experienced physicians and 7.7% of current residents/fellows (*p* = 0.001).

Family medicine physicians were more likely than psychiatrists to identify reasons such as lack of training or experience (PR: 2. 4, 95% CI: 1.58–3.65, *p* < 0.001), their preference to refer patients to Alcoholics Anonymous or another 12-step program (PR: 3.17, 95% CI: 1.20–8.35, *p* = 0.020), and concerns about the cost of these medications (PR: 6.67, 95% CI: 1.55–28.70, *p* = 0.011). Among the physicians who had not prescribed medications in treating AUDs, the majority were FM physicians (75.3%), and most identified lack of training or experience with these medications as the number one obstacle (*n* = 85, 70.8%).

### Promoting Use of Pharmacotherapy

A majority of all physicians sampled (87.6% of FM, 66.7% of psychiatrists) would increase their prescription of medications to treat AUDs if they had more training. FM physicians were more likely to use pharmacotherapy to treat AUDs if they had more time with their patients (PR: 2.29, 95% CI: 1.53–3.42, *p* < 0.001). Physicians’ preferred method of training was continuing medical education or Grand Rounds at their place of practice (*n* = 336, 70.7%), online training modules (56.8%), reading materials *via* email or mail (48.4%), and live workshops (41.1%).

## Discussion

In our sample, use of FDA-approved medications for treatment of alcohol dependence was the most common amongst both specialties, and psychiatrists had a greater tendency to prescribe medications without FDA approval. However, FM physicians were more likely to report lack of results with the medication options offered. In general, both specialties had concerns about the efficacy of all non-FDA-approved medications, but even among FDA-approved medications, physicians reported limited success in treatment.

A large proportion of physicians in our sample reported using antidepressants as treatment for AUDs. However, the literature supports no such use for alcohol dependence (17), unless comorbidity with depression is taken into consideration.

There are several limitations to this study. First, due to the sampling methodology, we were unable to calculate a survey response rate despite a large response. However, our data show similar trends in the prescribing practices of these two specialties, as seen in previous surveys found in the literature (6, 9, 14, 15). Second, due to the convenience sampling methodology employed, the findings reflected in this study may not be generalizable to all psychiatrists and FM specialists. Our sample included only a very small group of physicians with specialized training in addictions. Therefore, it is not possible to compare their responses regarding efficacy to those of physicians without specialized training. Previous studies in Veterans Health Administration facilities and specialized addiction treatment facilities, however, have noted that presence and availability of addictions-trained physicians increased the practice of pharmacotherapy in treatment of alcohol dependence (4, 7, 8).

When examining efficacy, our survey did not gather specific information about patient population, duration of treatment, or dosing. It is possible that physicians’ concerns about efficacy are related to their own understanding and expectations of efficacy of these medications for the treatment of AUDs. For example, in research trials, only relapse to heavy drinking was considered a true relapse in the analyses for the majority of medications promoting abstinence (naltrexone, acamprosate, and disulfiram). Drinking “slips” and “lapses” were not counted when determining efficacy in maintaining abstinence, and therefore, providers and/or patients may have had different expectations. Often patients quickly abandon medications they believe are not efficacious and do not allow for the prolonged therapeutic trial, as is the case in research studies. Although abstinence is the desired goal for most treatment approaches, even reduction in drinking has shown to reduce negative consequences and improve quality of life.

“Efficacy” was not defined in our study, and physicians of different specialties and/or levels of training may have had different metrics for determining efficacy. This difference in clinical significance is an important factor that has been found in other studies to matter even more to physicians than the demonstrated effect size of these medications (6, 16). Mark et al. found that physicians prescribed antidepressants to patients with alcohol dependence at a rate 3.5 times that of naltrexone, although they were able to correctly identify the effect size of antidepressants in treatment of depression as much smaller than that of naltrexone (6).

The survey assesses lifetime prescription of these medications, but not the proportion of patients with AUDs that these physicians treat. A physician with only a handful of patients with AUDs who are receiving pharmacotherapy may have a different perception than a physician with more experience in treating this population, which may affect perception of treatment response in some of the prescribers. A higher volume of patients with AUDs allows for more opportunities to use medications and experience success.

Among those physicians who have not used pharmacotherapy in the treatment of AUDs, with FM physicians being the majority, scant training was identified as the main problem impeding greater prescription. Similarly, physicians currently in training had lower prescription rates compared to senior physicians. Insufficient knowledge and clinical skills in the evaluation and management of SUDs among physicians in training can be exacerbated by negative attitudes toward this patient population (18).

A large proportion of physicians would welcome the opportunity of incorporating education in residency training programs on pharmacotherapy for AUDs. As in other surveys, some physicians selected increasing patient education about these medications as desirable, as it allows for a greater interest in trials of pharmacotherapy. Psychosocial treatment, including 12-step programs, and pharmacotherapy are not mutually exclusive and can be used simultaneously for better results. The COMBINE study revealed that the use of medications like naltrexone and acamprosate in combination with medical counseling (non-specialized) can have clinically significant outcomes, highlighting that delivery of effective treatment can occur in health-care settings where specialized addiction treatment is not available (19).

Determining the best predictors for treatment response should help alleviate some of the uncertainties reported with medications’ efficacy and would lead to a more individualized treatment approach. This would take into account pharmacogenetics, drinking patterns (11, 17), comorbidities, and treatment goals.

## Author Contributions

The three authors of this article had various kinds of roles for this perspective study. NA-D designed this study as well as contributed to the sections of the manuscript. CPM was responsible for the data collection, writing portions of the manuscript and helped in reviewing of existing literature. PV contributed to the data analysis section of the manuscript. The manuscript was finally reviewed by NA-D.

## Conflict of Interest Statement

The authors report no conflicts of interest. The authors alone are responsible for the content and writing of this paper.
